# Discriminative Mobility Characteristics between Neurotypical Young, Middle-Aged, and Older Adults Using Wireless Inertial Sensors

**DOI:** 10.3390/s21196644

**Published:** 2021-10-06

**Authors:** Clayton W. Swanson, Brett W. Fling

**Affiliations:** 1 Brain Rehabilitation Research Center, Malcom Randall VA Medical Center, 1601 SW Archer Road, Gainesville, FL 32608, USA; Clayton.Swanson@va.gov; 2 Department of Health & Exercise Science, Colorado State University, Fort Collins, CO 80521, USA; 3 Molecular, Cellular and Integrative Neuroscience Program, Colorado State University, Fort Collins, CO 80521, USA

**Keywords:** mobility, aging, middle-aged adults, young adults, older adults, gait, balance, sit-to-stand, turning, inertial sensors

## Abstract

Age-related mobility research often highlights significant mobility differences comparing neurotypical young and older adults, while neglecting to report mobility outcomes for middle-aged adults. Moreover, these analyses regularly do not determine which measures of mobility can discriminate groups into their age brackets. Thus, the current study aimed to provide a comprehensive analysis for commonly performed aspects of mobility (walking, turning, sit-to-stand, and balance) to determine which variables were significantly different and furthermore, able to discriminate between neurotypical young adults (YAs), middle-aged adults (MAAs), and older adults (OAs). This study recruited 20 YAs, 20 MAAs, and 20 OAs. Participants came into the laboratory and completed mobility testing while wearing wireless inertial sensors. Mobility tests assessed included three distinct two-minute walks, 360° turns, five times sit-to-stands, and a clinical balance test, capturing 99 distinct mobility metrics. Of the various mobility tests assessed, only 360° turning measures demonstrated significance between YAs and MAAs, although the capacity to discriminate between groups was achieved for gait and turning measures. A variety of mobility measures demonstrated significance between MAAs and OAs, and furthermore discrimination was achieved for each mobility test. These results indicate greater mobility differences between MAAs and OAs, although discrimination is achievable for both group comparisons.

## 1. Introduction

Typical aging throughout the lifespan results in a wide array of deleterious adaptations. A common maladaptation is the reduced capacity to perform activities of daily living because of impaired mobility. Coinciding reduced mobility, is the increased incidence of falls, which can lead to devastating repercussions, especially for individuals over the age of 65 [1,2]. While various mobility characteristics can help predict individuals at greater risk of falling [3,4,5,6,7,8,9,10,11], most age-related mobility research has focused on mobility differences between neurotypical young adults (e.g., ≤30 years of age) and their elderly (≥65 years) counterparts. For instance, older adults over the age of 65 often demonstrate reductions in spatiotemporal characteristics of mobility, such as walking speed, turn duration, sit-to-stand duration, and postural stability (i.e., greater path length), compared to young adults [2,8,12,13]. However, mobility adaptations and differences for those in the middle-aged years of life (30–65 years of age) have received far less attention to date. Moreover, many of the documented characteristics of mobility are quite general and non-specific and may not provide the most descriptive impairments for age-related mobility decline. Thus, there is a relative lack of research assessing which mobility characteristics can discriminate between the three primary adult life stages: young, middle, and older. Identifying mobility measures that are distinct to each life stage provides valuable insight as to the progression of mobility decline as a product of age and furthermore could highlight mobility characteristics associated with age-related pathologies [14].

An evolving body of research has focused on identifying mobility characteristics associated with disability, which has been instrumental in devising efficacious rehabilitation strategies and clinical biomarkers [15,16,17,18]. Moreover, with the advancement of inertial measurement unit (IMU) technology and algorithm development, the capacity to objectively quantify and track mobility has greatly improved, providing an abundance of reliable spatiotemporal metrics capable of characterizing numerous mobility domains and activities of daily living (i.e., walking, turning, balancing, etc.) [19,20]. While mobility differences between young adults and older adults remain insightful, recent research has begun to investigate which mobility tests and variables can discriminate within and between various populations [21,22,23]. In establishing discriminative mobility characteristics, clinicians and researchers can identify mobility assessments and measures capable of differentiating individuals between their neurotypical or atypical counterparts or identify distinct groups that demonstrate mobility similarities [22,24]. For instance, recent work assessed a multitude of gait, turning, and balance specific variables to determine which variable(s) or combination of variables were capable of discriminating between middle-aged people with multiple sclerosis (PwMS) and neurotypical middle-aged adult controls [22]. While many variables were significantly different between groups, it is interesting to note that discrimination was not solely influenced by the variables that achieved the greatest groupwise significant differences. For instance, while numerous gait variables demonstrated statistical significance between groups, single-limb support (% gait cycle time (%GCT)) provided the best discrimination between groups. For turning measures, five 360° turn variables demonstrated significant group differences, although excellent discrimination between groups was only achieved with two turning variables. Lastly, nearly all 32 balance variables for multiple balance conditions were significantly different between groups, although one particular balance condition provided the best discrimination between groups [22]. Therefore, to identify those demonstrating mobility disability, and further, develop efficacious rehabilitation approaches, it is important to identify which components of mobility categorize individuals into distinct groups.

Based on prior evidence highlighting kinematic differences between neurotypical young adults (YAs) and older adults (OAs), the focus of the current study was on comparing neurotypical YAs to middle-aged adults (MAAs), and MAAs to OAs. The objectives of the current study were to (1) identify whether objective measures of walking, turning, sit-to-stand, and balance were significantly different, and able to discriminate between neurotypical YAs, MAAs, and OAs; (2) identify whether three distinct walking conditions (self-selected normal pace, dual-task, and fast pace) influence discrimination between groups; and (3) determine whether a combination of task-specific mobility variables could better discriminate between groups while accounting for multicollinearity. As such, we hypothesized different levels of significance would be observed for gait, turning, sit-to-stand, and balance-related variables depending on the groups being compared. Specifically, for gait, we hypothesized that mean spatiotemporal lower body measures would demonstrate the greatest significance and discrimination between YAs and MAAs, while variability-related spatiotemporal measures would demonstrate the most significant and discriminative variables between MAAs and OAs. For 360° turning measures, we hypothesized that the most significant differences and discrimination would be associated with mean measures of turn duration, peak turn velocity, and turn angle for each group comparisons. For the five times sit-to-stand (5×STS), we hypothesized that YAs and MAAs would demonstrate similar spatiotemporal characteristics, while STS variables would be significantly different between MAAs and OAs, and further, provide discrimination between the two groups. For balance metrics, we hypothesized YAs and MAAs would demonstrate no significance and discriminatory capacity, while MAAs and OAs would demonstrate significant differences and discriminatory capacity particularly for the most challenging balance conditions. For objective two, we hypothesized that the dual-talk and fast walking conditions would produce greater levels of discrimination compared to the normal self-selected pace walking condition for both group comparisons. Lastly, for objective three, we hypothesized that greater discriminatory capacity would be observed when multiple variables were withheld in the stepwise regression for both group comparisons.

## 2. Materials and Methods

### 2.1. Participants

A total of 60 individuals separated into three distinct cohorts participated in the study and completed all gait and balance assessments (participant characteristics, Table 1). However, two participants (one YA, one MAA) withdrew their participation due to non-study-related reasons, and therefore did not complete the second day of testing, which assessed 360° turns and the 5×STS. Additionally, during analysis of the 5×STS test, three additional participants (one MA, two OA) recorded incomplete data, such that not all five sit-to-stand transitions were captured via the wireless inertial sensors or because of human error in counting the five transitions. While these five participants did not have complete data sets, their other mobility results were retained for other analyses. Participants included in this analysis were able to ambulate independently and free from any neurological disease or condition that would impact their mobility aside from the neurotypical effects of aging. This study was approved by the Colorado State University Institutional Review Board (18-7738H, 1/14/2019), and all participants provided written informed consent prior to their participation.

### 2.2. Procedures

Two days of data collection were conducted for each participant and separated by a minimum of 24 h and maximum of two weeks. For visit one, participants performed a series of three distinct two-minute walks, where they walked continuously back and forth down a well-lit 110-foot-long hallway. These walks included a normal self-selected pace, a dual-task, and a fast self-selected pace walk. For the dual-task condition, participants were asked to perform serial seven subtraction starting at a consistent pre-determined number. In addition, participants performed the modified Clinical Test of Sensory Interaction on Balance (CTSIB). The CTSIB incorporates four sequential balance trails designed to assess principal components of the balance system (i.e., vision, vestibular, and somatosensory) under different static balance conditions [25]. For each trial of the CTSIB, participants were asked to maintain their balance and stand as still as possible during the 30 s trial. For trial one, participants stood on a firm surface with their eyes open; for trial two, participants stood on a firm surface with their eyes closed; and trials three and four mimicked the first two trials, although the participants stood on a compliant foam surface (i.e., AIREX^®^ Balance-Pad).

For visit two, participants completed a series of three 360° in-place turns. For the 360° turns, participants were instructed to turn 360° clockwise with an immediate 360° turn counterclockwise. Participants were also asked to perform a 5×STS. For the 5×STS, participants were seated in a standardized chair (46 cm in height) without wheels or arm railings. They were asked to place their arms across their chest prior to trial initiation and maintain that arm position for the duration of the trial. Participants were then asked to perform five consecutive sit-to-stand movements at their self-selected fast but safe pace. Then, following the fifth sit-to-stand, participants were instructed to remain seated.

**Figure 1:**
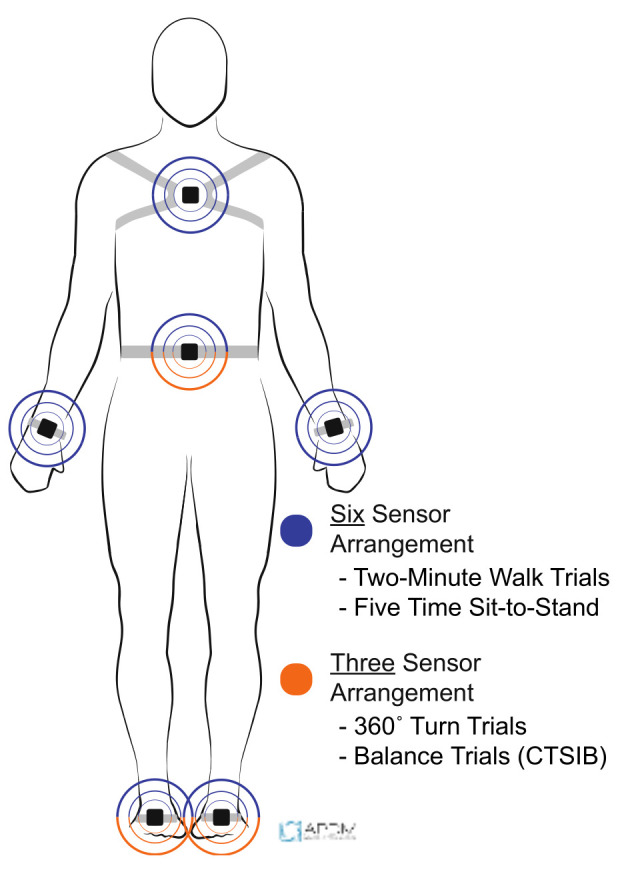
Six-sensor (blue) and three-sensor (orange) inertial sensor arrangement.

For both visits, participants performed all mobility tests unshod while wearing wireless inertial sensors. The wireless inertial sensors were placed in two standardized arrangements, either a six-sensor or three-sensor arrangement (Figure 1) (Opals V2, APDM, Inc., Portland, OR, USA). For the six-sensor arrangement, sensors were secured via elastic straps to the dorsum of each foot, around the waist at the L4-L5 level, on each wrist, and on the sternum; while the three-sensor arrangement placed sensors on the dorsum of each foot and around the waist at L4-L5. The six-sensor arrangement was used for the two-minute walk tests and the 5×STS test, while the three-sensor arrangement was used for the 360° turn test and the CTSIB.

Each wireless inertial sensor collects data at 128 Hz and houses an accelerometer, gyroscope, magnetometer, and barometer. Following each trial, data was wirelessly streamed from the wireless sensors to a laptop computer, where mobility variables were automatically quantified through Mobility Lab software (V2, APDM, Inc., Portland, OR, USA). The magnitude of variability was either quantified in Mobility Lab (i.e., lateral step variability) or calculated from the Mobility Lab output as the coefficient of variation (CoV) (SD/Mean).

### 2.3. Statistical Analysis

Based on the assumptions of the central limit theorem, parametric analyses were performed [26]. The Mobility Lab output provides both lower limb variables for each leg independently; therefore, we assessed leg-specific differences using a paired *t*-test. As none of the lower limb variables demonstrated significance, they were therefore averaged together for each gait variable. Likewise, the three independent 360° in-place turns did not demonstrate significance between trials and therefore, each 360° turn variable was averaged. All statistical analyses were performed using JMP Pro 15, with an alpha level set to 0.05. To assess differences between groups for demographics, 5×STS, and 360° in-place turns, we performed univariate analyses. For gait and balance measures, separate repeated measure analysis of variance (RMANOVA) was performed. Specifically, for gait measures, three 2 × 3 RMANOVA were performed with post-hoc analyses assessing significant interactions. For balance measures, three 2 × 4 RMANOVA were performed with post-hoc analyses for significant interactions.

To examine the measures of mobility and which variables were capable of best discriminating between groups (YAs–MAAs and MAAs–OAs), the receiver operating characteristic (ROC) curve and the area under the curve (AUC) were calculated for all mobility variables. Since gait and balance performance were each measured under multiple conditions, the ROC and AUC were calculated for each condition and variable separately. For classification, an AUC of 0.50 denoted no discriminatory ability, 0.70–0.79 was considered acceptable, 0.80–0.89 was considered excellent, and ≥0.90 was considered outstanding [27,28]. For the following analysis, an AUC threshold of 0.80 was established, denoting excellent discrimination between groups and the corresponding variable(s).

Upon establishment of AUC values for each mobility metric, the top five AUC producing variables (independent of meeting the 0.80 threshold) from each mobility test (e.g., two-minute self-selected pace walk, dual-task walk, fast walk, 360° turning, 5×STS, etc.) were placed into a backwards stepwise regression. The stepwise regression was incorporated to help identify the best fit model using the lowest Akaike information criterion (AICc) value. This analysis was used as it accounts for multicollinearity and identifies potential predictor variables capable of describing variance and discrimination between paired groups.

## 3. Results

In total, 99 independent metrics were collected from the four mobility tests. Due to the inertial sensors housing both an accelerometer and gyroscope, certain variables display similar names despite being independent measures (see Appendix A, Table A1 for variable definitions).

### 3.1. Two-Minute Walk Measures

#### 3.1.1. Gait and 180° Turning Measures between Neurotypical Young Adults and Middle-Aged Adults

**Figure 2:**
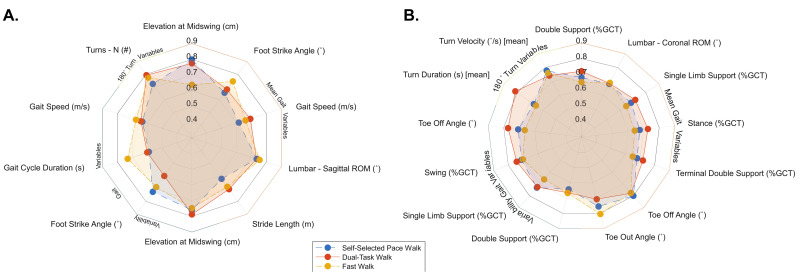
Spider plot indicating the variables that were consistently observed in the top 20 AUC producing variables for each walking condition. (**A**) Represents the 10 AUC producing variables that were observed in all three walking conditions between YAs and MAAs. (**B**) Represents the 13 AUC producing variables that were observed in all three walking conditions between MAAs and OAs.

Of the combined 52 gait and 180° turning variables, none demonstrated a group × condition interaction, although for the independent variable AUC analysis, mean elevation at midswing (cm) did achieve the 0.80 discriminatory threshold for the normal self-selected pace walking condition. No other variables from the three walking conditions met the threshold independently, although several variables from each walking condition achieved ‘acceptable’ (≥0.70–≤0.79) discriminatory ability (Table 2). For the normal self-selected pace stepwise regression, two variables (elevation at midswing (CoV) and stride length (CoV)) were withheld in the regression, together achieving outstanding discrimination (AUC = 0.93). For the dual-task walking condition, again two variables were withheld (mean elevation at midswing (cm) and number of turns (#)), and together achieved outstanding discrimination (AUC = 0.92). Lastly, for the fast-walking condition, three variables (elevation at midswing (CoV), number of turns (#), cadence (CoV)) were withheld, achieving outstanding discrimination (AUC = 0.94). Figure 2A displays the independent gait metrics that were not only a top-20 AUC producing variable, but also consistently placed in the top 20 for each walking condition.

#### 3.1.2. Gait and 180° Turning Measures between Neurotypical Middle-Aged Adults and Older Adults

Of the combined 52 gait and 180° turning variables, four variables (mean trunk–transverse range of motion (ROM) (°), mean lumbar–sagittal ROM (°), lumbar–coronal ROM (CoV), and toe off angle (CoV)) demonstrated a significant group × condition interaction (Table 3). Although, the post-hoc analysis for the group × condition interaction only maintained significance for certain walking conditions for three of the gait variables (Appendix B, Table A2). For mean lumbar–sagittal ROM (°) in the fast-walking condition, OAs demonstrated significantly reduced lumbar ROM in the sagittal plane, with neither of the other walking conditions demonstrating significance. For lumbar–coronal ROM (CoV) in the dual-task condition, OAs demonstrated significantly increased coronal ROM variability, with no other walking conditions demonstrating significance. Lastly, for toe off angle (CoV) during the normal and dual-task walking conditions, OA exhibited significantly increased toe off angle variability, while the fast-walking condition did not demonstrate post-hoc significance.

For the condition x group post-hoc analysis, no significant differences were revealed for mean trunk–transverse ROM (°) for either group. Although MAA demonstrated significant differences for mean lumbar–sagittal ROM (°) between the normal and fast-paced walks and between the dual-task and fast-paced walks, OA did not demonstrate significant differences between any of the walking conditions. MAA also demonstrated significant differences between the dual-task and fast walking conditions for lumbar–coronal ROM (CoV), while OA did not demonstrate significance between any walking condition. Lastly, for toe off angle (CoV), MAA did not demonstrate any differences between walking conditions; however, OA did demonstrate significance between the dual-task walk and fast-walking conditions (Appendix B, Table A3).

The AUC analysis for each condition provided one variable that met the AUC threshold. Specifically, for the normal walking condition, mean toe off angle (°) produced an AUC of 0.80, denoting excellent discriminatory ability. Five additional variables for the normal walking condition demonstrated acceptable (≥0.70) discriminatory ability. For the dual-task walking condition, mean turn duration (s) demonstrated excellent discriminatory ability (AUC = 0.81), while 15 addition variables encompassing mean linear gait, turning, and variability measures provided acceptable discrimination (AUC ≥ 0.70). Lastly, for the fast-walking condition, mean toe out angle (°) alone demonstrated excellent discriminatory ability with an AUC of 0.81, with seven additional variables demonstrating acceptable discrimination.

For the normal self-selected pace walking condition stepwise regression analysis, three variables were maintained in the model. Specifically, this included mean toe off angle (°), mean toe out angle (°), and single limb support (%GCT) (CoV). Together, these variables produced an AUC of 0.94, demonstrating outstanding discriminatory ability between MAA and OAs. For the dual-task walking condition, mean turn duration (s) and swing (%GCT) (CoV) were maintained in the stepwise regression, producing an excellent discriminatory ability (AUC = 0.84). Lastly, for the fast-walking condition, mean toe out angle (°) and mean toe off angle (°) produced excellent discriminatory ability (AUC = 0.85). Figure 2B displays the independent AUC values for those that were in the top 20 and overlapped across the three walking conditions.

### 3.2. 360° Turning Measures

#### 3.2.1. 360° Turning Measures between Neurotypical Young Adults and Middle-Aged Adults

**Figure 3:**
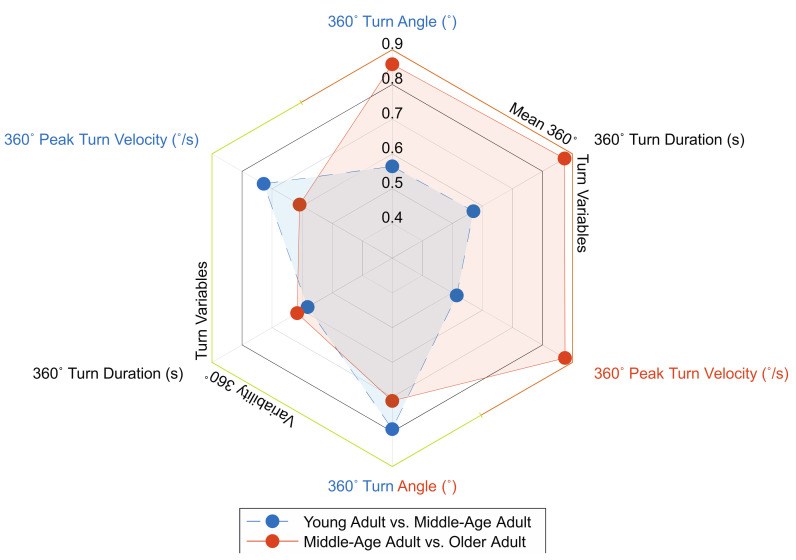
Spider plot denoting each 360° turning variable and their associated AUC values between groups. Colored variables represent those maintained in the stepwise regression model for each group comparison.

Two of the 360° turn variables demonstrated significance between YAs and MAAs, with no variables independently meeting the 0.80 AUC threshold. Although, 360° turn angle variability nearly met the threshold as an independent turn variable (AUC = 0.79) (Table 4, Figure 3). The stepwise regression withheld three of the turn variables, subsequently achieving an AUC of 0.89. Thus, this denoted excellent discriminatory ability between groups for those combined turning metrics (Table 4). These results indicate that when mean and variability-related 360° turn measures are combined in a regression model, they provide greater discriminatory ability when compared to independent 360° turn measures.

#### 3.2.2. 360° Turning Measures between Neurotypical Middle-Aged Adults and Older Adults

Four 360° turn variables demonstrated significance between MAAs and OAs, with three turning variables independently reaching the discriminatory threshold (Table 5, Figure 3). The stepwise regression withheld two 360° turning variables, achieving outstanding discriminatory ability for those variables maintained in the model (AUC = 0.94). These results indicate that while multiple 360° turn variables independently are capable of meeting an excellent discriminatory threshold, the stepwise regression model provides greater discriminatory capacity.

### 3.3. Sit-to-Stand Measures

#### 3.3.1. Sit-to-Stand Measures between Neurotypical Young Adults and Middle-Aged Adults

**Figure 4:**
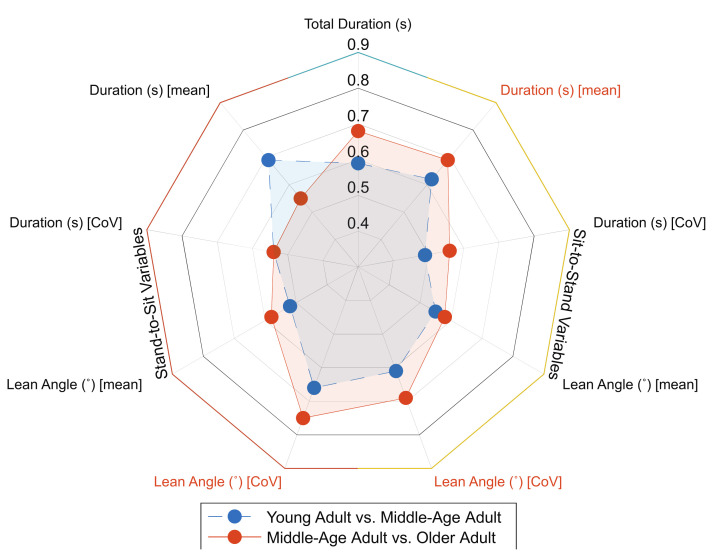
Spider plot representing all STS variables and their associated AUC values between groups. The variable names in color are those that were maintained in the stepwise regression for the group comparison.

None of the 5×STS variables demonstrated significance between groups. Additionally, none of the variables independently or as a product of the stepwise regression met the AUC threshold (Table 6, Figure 4). For the stepwise regression, none of the variables included in the model were retained, such that the AICc for all possible models were greater than that of the intercept.

#### 3.3.2. Sit-to-Stand Measures between Neurotypical Middle-Aged Adults and Older Adults

Two 5×STS variables demonstrated significant differences between MAAs and OAs. For the AUC analysis, no variables independently met the AUC threshold, although stand-to-sit lean angle variability demonstrated acceptable discrimination (Table 7, Figure 4). When including the top five AUC producing variables, two were maintained in the model, which together demonstrated excellent discriminatory ability (AUC = 0.86). These results indicate that while none of the variables independently met the AUC threshold, multiple variables combined achieved excellent discrimination.

### 3.4. Balance Variables

#### 3.4.1. Balance Variables between Neurotypical Young Adults and Middle-Aged Adults

**Figure 5:**
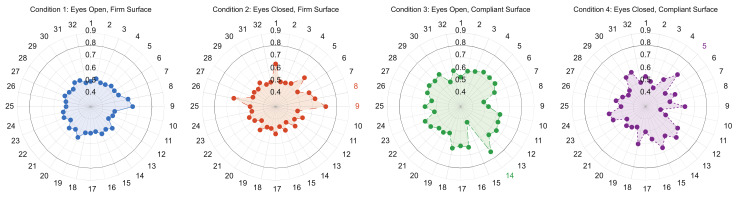
Spider plot representing all balance variables assessed and their linked AUC value between YA and MAA groups. Colored variable numbers round the spider plot indicate variable(s) withheld in the stepwise regression for each condition. Numbers and corresponding variable names: 1—95% Ellipse Radius (rad), 2—95% Ellipse Axis 1 Radius (m/s^2^), 3—95% Ellipse Axis 2 Radius (m/s^2^), 4—Centroidal Frequency (Hz), 5—Centroidal Frequency (Coronal) (Hz), 6—Centroidal Frequency (Sagittal) (Hz), 7—Frequency Dispersion (AD), 8—Frequency Dispersion (Coronal) (AD), 9—Frequency Dispersion (Sagittal) (AD), 10—Jerk (m^2^/s^5^), 11—Jerk (Coronal) (m^2^/s^5^), 12—Jerk (Sagittal) (m^2^/s^5^), 13—Mean Velocity (m/s), 14—Mean Velocity (Coronal) (m/s), 15—Mean Velocity (Sagittal) (m/s), 16—Path Length (m/s^2^), 17—Path Length (Coronal) (m/s^2^), 18—Path Length (Sagittal) (m/s^2^), 19—Range (m/s^2^), 20—Range (Coronal) (m/s^2^), 21—Range (Sagittal) (m/s^2^), 22—RMS Sway (m/s^2^), 23—RMS Sway (Coronal) (m/s^2^), 24—RMS Sway (Sagittal) (m/s^2^), 25—Sway Area (m^2^/s^4^), 26—95% Ellipse Radius (°), 27—95% Ellipse Axis 1 Radius (°), 28—95% Ellipse Axis 2 Radius (°), 29—RMS Sway (°), 30—RMS Sway (Coronal) (°), 31—RMS Sway (Sagittal) (°), 32—Sway Area (°^2^).

None of the 32 postural stability variables revealed a significant group × condition interaction. Moreover, no variable independently met the AUC threshold, nor did the stepwise regression produce a model capable of discrimination for any balance condition (Table 8, Figure 5). These results indicate postural stability similarities for each condition of the CTSIB between YAs and MAAs.

#### 3.4.2. Balance Variables between Neurotypical Middle-Aged Adults and Older Adults

Fourteen of the 32 sway variables revealed a significant group × condition interaction. Although, no significant differences for conditions 1 (eyes open, firm surface) or 2 (eyes closed, firm surface) were observed in the post-hoc analysis. Condition 3 (eyes open, compliant surface) demonstrated significant group differences for seven balance variables. Condition 4 (eyes closed, compliant surface) revealed significance between groups for 13 balance variables (Appendix C, Table A4). The condition × group post-hoc analysis demonstrated no significant differences between conditions 1 and 2 for either group. Although, the comparisons between condition 4 (eyes closed, compliant surface) and the other three balance conditions demonstrated significance between MAA and OA for most sway variables (Appendix C, Table A5).

**Figure 6:**
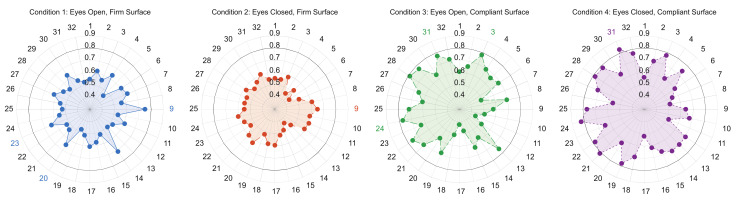
Spider plot representing all balance variables assessed and their linked AUC value between MAA and OA groups. Colored variable numbers round the spider plot indicate variable(s) withheld in the stepwise regression for each condition. Numbers and corresponding variable names: 1—95% Ellipse Radius (rad), 2—95% Ellipse Axis 1 Radius (m/s^2^), 3—95% Ellipse Axis 2 Radius (m/s^2^), 4—Centroidal Frequency (Hz), 5—Centroidal Frequency (Coronal) (Hz), 6—Centroidal Frequency (Sagittal) (Hz), 7—Frequency Dispersion (AD), 8—Frequency Dispersion (Coronal) (AD), 9—Frequency Dispersion (Sagittal) (AD), 10—Jerk (m^2^/s^5^), 11—Jerk (Coronal) (m^2^/s^5^), 12—Jerk (Sagittal) (m^2^/s^5^), 13—Mean Velocity (m/s), 14—Mean Velocity (Coronal) (m/s), 15—Mean Velocity (Sagittal) (m/s), 16—Path Length (m/s^2^), 17—Path Length (Coronal) (m/s^2^), 18—Path Length (Sagittal) (m/s^2^), 19—Range (m/s^2^), 20—Range (Coronal) (m/s^2^), 21—Range (Sagittal) (m/s^2^), 22—RMS Sway (m/s^2^), 23—RMS Sway (Coronal) (m/s^2^), 24—RMS Sway (Sagittal) (m/s^2^), 25—Sway Area (m^2^/s^4^), 26—95% Ellipse Radius (°), 27—95% Ellipse Axis 1 Radius (°), 28—95% Ellipse Axis 2 Radius (°), 29—RMS Sway (°), 30—RMS Sway (Coronal) (°), 31—RMS Sway (Sagittal) (°), 32—Sway Area (°^2^).

No variables in conditions 1, 2, or 3 met the AUC threshold independently, although condition 3 had multiple variables that demonstrated an acceptable AUC threshold (AUC ≥ 0.70). Condition 4 was the only condition that produced independent variables that met the AUC threshold. Specifically, three variables associated with the sagittal plane independently met the threshold (Table 9). The stepwise regression that included the top five AUC producing variables for each condition produced models for conditions 1, 3, and 4 that achieved excellent discrimination (Table 9). Specifically, for condition 1, three sway metrics (frequency dispersion (sagittal) (AD), range (coronal) (m/s^2^), and RMS sway (coronal) (m/s^2^)) were withheld and produced an AUC of 0.84. For condition 3, three sway metrics (95% ellipse axis 2 radius (m/s^2^), RMS sway (sagittal) (m/s^2^), and RMS sway (sagittal) (°)) were withheld and produced an AUC of 0.87, and lastly, for condition 4, one sway measure (RMS sway (sagittal) (°)) was withheld, maintaining an AUC of 0.83. Figure 6 displays all the independently quantified balance metrics and their associated AUC values for each condition.

## 4. Discussion

The primary objectives of this study were to identify whether measures of gait, turning, sit-to-stand, and balance were significantly different and able to discriminate between neurotypical YAs and MAAs and MAAs and OAs. Additionally, we set out to determine whether different walking conditions (self-selected pace, dual-task, fast pace) modify discriminatory capacity, and lastly assessed whether the five highest AUC achieving variables for each mobility measure could provide enhanced discrimination between paired groups. Between YAs and MAAs, our results highlight excellent discriminatory capacity for combined gait and turning measures, although widespread mobility similarities (i.e., non-significant differences between variables) for all mobility tests were observed, especially gait, STS, and balance measures. The comparison between MAA and OA resulted in numerous variables for each mobility test, which demonstrated significant differences between groups and furthermore, excellent to outstanding ability to discriminate between groups. 

### 4.1. Mobility between Neurotypical Young Adults and Middle-Aged Adults

Of the 99 mobility measures collected and assessed, only two variables demonstrated significance between YAs and MAAs, despite the 21-year age difference. These results support prior research demonstrating gait-related similarities between YAs and MAAs, although to our knowledge, this is the first study to comprehensively assess multiple mobility tests between these distinct groups. Of the various gait metrics assessed, none demonstrated significance between groups or walking conditions. These results coincide with prior investigations showing no differences in traditionally measured spatiotemporal metrics for self-selected speed or between single- and dual-task walking performance measures [2,29,30,31,32]. While studies have reported gait similarities between YAs and MAAs, two studies have revealed differences between similarly aged groups for measures of gait variability. Specifically, stride width variability and center of pressure (CoP) variability have demonstrated differences, such that MAAs show increased variability [33,34]. Although no gait variables for any condition demonstrated significant differences in the current study, the AUC analysis did reveal the ability to discriminate between groups both independently and as a result of the stepwise regression. Specifically, mean elevation at midswing (cm) independently met the AUC threshold for excellent discriminatory ability for the self-pace normal walking condition. Moreover, the stepwise regression through a combination of variables provided outstanding discriminatory ability for each walking condition. Interestingly, elevation at midswing variability was consistently withheld for each walking condition and number of turns completed was withheld for the dual-task and fast-walking conditions. While more research is necessary, these results highlight lower limb variability and turning measures as important indicators of discrimination between YAs and MAAs independent of statistical significance between groups.

Of the six 360° turn variables assessed, two variability measures (turn angle (°) and peak turn velocity (°/s)) demonstrated significance between groups, contrary to our original hypothesis, which suggested that mean turning measures would be the variables to demonstrate significance. Given the dynamic nature of 360° turns, these results may partially be explained by the previously reported increased variability of CoP during gait by Bizovska et al. (2014), although further research is warranted [34]. While turn angle variability nearly met the AUC threshold, none of the variables independently met the threshold. As for the stepwise regression, a combination of mean and variability measures were withheld, achieving excellent discriminatory ability. These results indicate an ability to delineate between YAs and MAAs using 360° turn measures.

In agreement with our hypothesis, none of the nine STS measures demonstrated any significance between groups or met the AUC threshold independently or as a product of the stepwise regression. These results signal that the spatiotemporal variables associated with STS movement collected as part of this analysis are not only similar between groups, but are also unable to provide discriminatory capacity.

In terms of balance, the group × condition interaction analysis revealed no significant differences for any measure, which is in agreeance with our hypothesis. Moreover, these results coincide with prior research indicating postural stability similarities for conditions 2 (eyes closed, firm surface) and 4 (eyes closed, compliant surface) [35,36]. Although differences have also been reported, for instance, CoP amplitude during conditions 1 (eyes open, firm surface) and 3 (eyes open, compliant surface) and CoP velocity for condition 3 were shown to be significantly greater in the MAA groups compared to the YA group [35]. The inconsistency between results could be associated with the collection protocol, for instance, Abrahamová et al. (2008) performed 50 s trials compared to our 30 s balance trials [35], possibly indicating that sensory weighing of vision is influenced as a product of longer trial lengths. Further, our results demonstrated no independent balance variable or combination of variables that met the AUC threshold, proposing that static postural stability appears to be comparable between YAs and MAAs, especially for the eyes closed balance conditions. Moreover, these results may suggest similar compensatory reliance strategies for maintenance of balance, such that their reliance on the various systems (vision, somatosensory, vestibular) is similarly weighted when performing these four conditions over the course of 30 s.

While these two groups have a 21-year age difference, the results demonstrated broad statistical similarities between mobility measures, such that only two of the 99 measured variables were significantly different between groups. While only two variables demonstrated significance, excellent discrimination was achievable for variables associated with walking and turning, indicating that trials associated with dynamic rather than static mobility are better able to discriminate between YAs and MAAs. Moreover, these results may indicate that while movement similarities are present, nuances within variables exist that can distinguish between these similarly performing groups.

### 4.2. Mobility between Neurotypical Middle-Aged Adults and Older Adults

The comparison between MAAs and OAs identified numerous mobility tests and variables that demonstrated both significance and discriminatory ability. Specific to gait, three variables demonstrated a significant interaction between groups, although none of them were both significant and met the AUC threshold. Two of the variables that demonstrated significance were associated with lumbar ROM in both the coronal and sagittal planes, such that OAs demonstrated increased coronal (medial-lateral) ROM, with reduced sagittal (anterior-posterior) ROM. Additionally, OAs demonstrated greater toe off angle variability compared to YAs. In terms of more conventional gait characteristics, our results are in agreeance with prior studies showing gait speed and stride length similarities between MAAs and OAs [37]. While toe off angle variability and lumbar (coronal and sagittal) ROM were significantly different between groups, those measures did not meet the discriminatory threshold. Although, three other variables did independently meet the discriminatory threshold without demonstrating a group difference. Specifically, mean toe off angle (°) during the normal self-selected pace walk, turn duration (s) during the dual task walk, and toe out angle (°) during the fast-walking condition independently met the AUC threshold. Interestingly, mean turn duration (s) during the dual-task condition achieved an AUC of 0.81, which is identical to a recently published study by Shah et al. (2020), who compared similarly aged neurotypical groups [21]. While the discriminatory capacity was identical, the study designs were quite different, for instance, the current study assessed gait during three distinct two-minute walking conditions while Shah and colleagues assessed gait in the free-living environment over the course of one week [21]. Regardless of the study design differences, however, mean turn duration (s) in both studies met the discriminatory threshold, possibly indicating that navigating the free-living environment is similar to performing a dual-task paradigm, particularly when performing turns. While turn duration between the two studies was discriminative, results from other gait variables did differ. For instance, both mean toe off angle (°) and toe out angle (°) (normal self-selected pace and fast-paced walking trials, respectively) independently met the AUC threshold in the current study, while Shah et al. (2020) reported a non-discriminatory value for toe off angle (°) (AUC = 0.51) and did not report values for toe out angle (°). We believe this difference could be due to study design differences, such that discrimination was observed during the normal self-selected pace walk while Shah and colleagues likely assessed toe off angle (°) during both linear and non-linear components of gait and naturally occurring gait speed fluctuations, which occur outside in the free-living environment. Although this interpretation is speculative, more research is needed to identify the similarities and differences between the free-living environment and various laboratory-controlled walking paradigms. While independent variables met the AUC threshold, the discriminatory capacity was improved via stepwise regression for each walking condition, with the normal self-selected pace walking condition achieving outstanding discriminatory capacity (AUC = 0.94). Since significance and discrimination may be achieved via different gait variables, these results highlight that multiple types of analyses should be performed.

Of the various 360° turning measures, four variables demonstrated significance between groups. Moreover, the three mean turning measures (turn angle (°), duration (s), and peak turn velocity (°/s)) independently demonstrated excellent discriminatory ability. Although, the stepwise regression achieved outstanding discrimination between groups through retention of the 360° turn angle variability and mean peak turn velocity (°/s). These results suggest that while the mean 360° turning measures are capable of independently discriminating between groups, the combination of mean and variability 360° turn measures improves the ability to discriminate between groups. In fact, this combination of 360° mean and variability turning metrics has been shown to improve discrimination between people with multiple sclerosis and age-matched neurotypical controls in a similar discriminatory analysis [22].

Of the nine STS variables, two demonstrated significant group differences. Specifically, the variability of sit-to-stand lean angle (°) and stand-to-sit lean angle (°) was significantly different between MAAs and OAs. The variability of lean angle during the sit-to-stand phase is consistent with prior investigations between healthy YAs and OAs [38], although to our knowledge, no research has assessed these particular metrics between MAAs and OAs. While lean angle variability demonstrated significance between groups, no variables independently met the discriminatory threshold. However, the combination of sit-to-stand duration (s), lean angle variability (°), and stand-to-sit lean angle variability together did achieve the discriminatory threshold, again indicating that the combination of mean and variability kinematics together improve discriminatory ability.

Of the various balance measures assessed, no variables in conditions 1 (eyes open, firm surface) or 2 (eyes closed, firm surface) demonstrated significance between groups, although seven variables in condition 3 (eyes open, compliant surface) and 13 variables in condition 4 (eyes closed, compliant surface) revealed group differences, with OA demonstrating reduced postural stability. These results agree with our hypothesis, which hypothesized that increasingly challenging balance conditions would demonstrate group differences. Additionally, these results align with prior research indicating significant differences between MAAs and OAs for balance conditions performed on a compliant surface. For instance, Low Choy et al. (2003) demonstrated quantifiable balance deficits between middle-aged and older women, which was amplified upon the introduction of a compliant surface [36], suggesting reduced sensory involvement of the vestibular and/or somatosensory systems, and an increased reliance on the visual system, which is thought to manifest during the fifth decade of life [36]. In addition to demonstrating statistical differences between groups, several variables associated with condition 4 independently met the AUC threshold, although the stepwise regression for condition 3 revealed the best discriminatory ability between all conditions. These results further corroborate reduced somatosensory feedback observed in OA, and provide commonly measured balance variables capable of discriminating between groups [39]. Taken together, both static and dynamic mobility tests demonstrated significance between MAAs and OAs, although not all significant variables demonstrated the ability to discriminate between groups. Interestingly, the stepwise regression for the normal pace self-selected walk and the 360° turn measures were able to provide the highest levels of discrimination, which may indicate that OAs have greater impairments associated with dynamic rather than static mobility.

A couple limitations of the current study need to be considered. First, discrimination between groups was based on the characterization of AUC values for each mobility test and measure, but it cannot be assumed the same outcomes would be observed for all YA, MAA, and OA cohorts. Future work incorporating larger cohorts of individuals is needed to identify whether these findings are generalizable to the various age groups. Second, the sex distribution was heavily weighted towards females for MAAs, and this could present mobility differences based on inherent anthropometric differences. However, our results demonstrated similar mobility outcomes and similar significance when compared to previously published results for sex-matched groups [30,37]. Although, it should be noted there is conflicting evidence documenting kinematic sex differences between sexes independent of age [40] but also as a product of age [41]; therefore, additional research should be performed to assess age- and sex-related discriminative mobility differences.

## 5. Conclusions

Together, our results demonstrate broad mobility similarities between YAAs and MAAs, although vast differences between MAAs and OAs indicate that mobility decline likely initiates near the fifth decade of life and continues to decline with age. While there was a lack of statistical significance for nearly all mobility variables between YAs and MAAs, there was the capability for non-significant variables to discriminate between cohorts. Particularly, discrimination was greater for dynamic (i.e., gait and turning) mobility tests rather than static (i.e., balance) mobility tests. Specifically, gait-related measures associated with variability and number of turns produced outstanding levels of discrimination while STS and balance measures did not produce variables capable of meeting the AUC threshold. This indicates that kinematic variability may be a precursor to age-related spatiotemporal movement deficits. Conversely, the comparison between MAA and OA produced statistical models capable of discrimination for each mobility measure, indicating that both dynamic and static components of mobility are influenced as a product of neurotypical aging. Though further investigation is needed, we believe these results highlight the need to assess mobility through multiple analytical approaches rather than merely reporting statistical significance, as it may provide additional prospective for greater interpretation. Additionally, these results identify variables capable of discriminating between three neurotypically healthy cohorts, which could be beneficial for detecting the early stages of mobility abnormalities that may lead to pathological identification.

## Figures and Tables

**Table 1:** Groupwise participant characteristics and demographics. *f*-statistics (*F*) and *p*-values (*p*) are reported for each group comparison. Where applicable, corresponding data is presented as means ± SD. BMI—Body Mass Index.

				Young Adults vs. Middle-Aged Adults		Middle-Aged Adults vs. Older Adults	
	Young Adults	Middle-Aged Adults	Older Adults	*F*	*p*	*F*	*p*
Number	20	20	20	-	-	-	-
Age (yrs)	25.1 ± 3.38	46.3 ± 10.83	70.5 ± 5.87	69.67	<0.001	77.54	<0.001
Sex	10 f (10 m)	15 f (5 m)	9 f (11 m)	-	-	-	-
Weight (kg)	67.5 ± 12.21	73.6 ± 13.56	77.0 ± 17.49	2.20	0.15	0.47	0.50
Height (cm)	171.5 ± 8.55	168.7 ± 7.88	170.9 ± 11.76	1.15	0.29	0.50	0.49
BMI	22.8 ± 2.56	25.8 ± 4.28	26.1 ± 3.83	7.24	0.01	0.04	0.85

**Table 2:** Top five AUC producing gait variables for each walking condition, their associated means ± SD, RMANOVA group by condition interaction *p*-value, independent AUC (I-AUC) value, and stepwise regression AUC (S-AUC) value. Variable(s) with a single (*) were included in the stepwise regression, those with a (**) were maintained in the regression for further AUC assessment and discriminatory ability. COV—coefficient of variation, ROM—range of motion.

		Young Adults		Middle-Aged Adults				
Gait Variables	Condition	Mean	SD	Mean	SD	Interaction	I-AUC	S-AUC
Elevation at Midswing (cm) [CoV] **	Normal	0.60	0.11	0.46	0.14	0.58	0.76	0.93
Elevation at Midswing (cm) [mean] *		0.47	0.20	0.81	0.37	0.55	0.80	
Foot Strike Angle (°) [CoV] *		0.07	0.02	0.09	0.03	0.40	0.73	
Lumbar-Sagittal ROM (°) [mean] *		4.32	1.26	5.35	1.62	0.23	0.74	
Stride Length (m) [CoV] **		0.02	0.00	0.03	0.01	0.55	0.74	
Elevation at Midswing (cm) [CoV] *	Dual Task	0.64	0.14	0.47	0.16	0.58	0.79	0.92
Elevation at Midswing (cm) [mean] **		0.40	0.15	0.67	0.31	0.55	0.78	
Lumbar-Sagittal ROM (°) [mean] *		4.01	1.16	4.85	1.39	0.23	0.73	
Stride Length (m) [mean] *		1.24	0.10	1.17	0.10	0.25	0.70	
Turns-N (#) **		4.35	0.75	3.50	0.61	0.41	0.79	
Cadence (steps/min) [CoV] **	Fast	0.02	0.00	0.03	0.01	0.17	0.76	0.94
Elevation at Midswing (cm) [CoV] **		0.61	0.14	0.48	0.13	0.58	0.75	
Foot Strike Angle (°) [mean] *		23.49	2.98	20.82	3.38	0.15	0.75	
Lumbar-Sagittal ROM (°) [mean] *		5.73	1.75	7.37	1.83	0.23	0.76	
Turns-N (#) **		6.65	0.75	5.75	0.91	0.41	0.78	

**Table 3:** Top five AUC producing gait variables for each walking condition, their associated means ± SD, RMANOVA group by condition interaction *p*-value, independent AUC (I-AUC) value, and stepwise regression AUC (S-AUC) value. Variable(s) with a single (*) were included in the stepwise regression, those with a (**) were maintained in the regression for further AUC assessment and discriminatory ability. %GCT—percent gait cycle time, COV—coefficient of variation, ROM—range of motion.

		Middle-Aged Adults		Older Adults				
Gait Variables	Condition	Mean	SD	Mean	SD	Interaction	I-AUC	S-AUC
Single Limb Support (%GCT) [CoV] **	Normal	0.02	0.01	0.02	0.01	0.17	0.73	0.94
Swing (%GCT) [CoV] *		0.02	0.01	0.02	0.01	0.19	0.71	
Toe Off Angle (°) [mean] **		39.80	4.48	33.84	5.38	0.61	0.80	
Toe Out Angle (°) [mean] **		7.65	5.95	13.42	6.57	0.17	0.76	
Turn Velocity (°/s) [mean] *		211.11	28.99	178.45	30.56	0.34	0.78	
Swing (%GCT) [CoV] **	Dual Task	0.02	0.01	0.02	0.01	0.19	0.74	0.84
Toe Off Angle (°) [CoV] *		0.03	0.01	0.05	0.02	0.03	0.77	
Toe Off Angle (°) [mean] *		38.31	4.82	32.67	5.74	0.61	0.78	
Turn Duration (s) [mean] **		2.03	0.20	2.41	0.42	0.06	0.81	
Turn Velocity (°/s) [mean] *		206.42	28.78	176.60	40.72	0.34	0.74	
Foot Strike Angle (°) [mean] *	Fast	20.82	3.38	17.52	4.67	0.70	0.71	0.85
Lumbar-Sagittal ROM (°) [mean] **		7.37	1.83	5.45	2.05	0.004	0.79	
Toe Off Angle (°) [mean] **		42.11	3.90	36.47	5.99	0.61	0.78	
Toe Out Angle (°) [mean] **		5.43	5.58	12.46	6.59	0.17	0.81	
Turn Velocity (°/s) [mean] *		271.80	49.25	223.08	49.88	0.34	0.76	

**Table 4:** Means, standard deviations, significance (*p*), independent AUC (I-AUC), and stepwise AUC (S-AUC) measures for 360° turning variables. Variable(s) with a single (*) were included in the stepwise regression, those with a (**) were maintained in the regression model for further AUC assessment and discriminatory ability.

	Young Adults		Middle-Aged Adults				
360° Turning Variable	Mean	SD	Mean	SD	*p*	I-AUC	S-AUC
360° Turn Angle (°) [Mean] **	384.89	11.86	385.04	9.15	0.97	0.56	0.89
360° Turn Angle (°) [CoV] **	0.01	0.01	0.02	0.01	<0.001	0.79	
360° Turn Duration (s) [Mean] *	1.91	0.30	1.90	0.35	0.91	0.57	
360° Turn Duration (s) [CoV] *	0.06	0.04	0.04	0.02	0.16	0.58	
360° Peak Turn Velocity (°/s) [Mean]	371.03	67.62	361.32	63.78	0.66	0.51	
360° Peak Turn Velocity (°/s) [CoV] **	0.07	0.03	0.05	0.02	0.009	0.73	

**Table 5:** Means, standard deviations, significance (*p*), independent AUC (I-AUC), and stepwise AUC (S-AUC) measures for 360° turning variables. Variable(s) with a single (*) were included in the stepwise regression, those with a (**) were maintained in the regression model for further AUC assessment and discriminatory ability.

	Middle-Aged Adults		Older Adults				
360° Turning Variable	Mean	SD	Mean	SD	*p*	I-AUC	S-AUC
360° Turn Angle (°) [Mean] *	385.04	9.15	370.37	10.58	<0.0001	0.86	0.94
360° Turn Angle (°) [CoV] **	0.02	0.01	0.01	0.01	0.01	0.71	
360° Turn Duration (s) [Mean] *	1.90	0.35	2.87	1.07	<0.001	0.87	
360° Turn Duration (s) [CoV] *	0.04	0.02	0.06	0.04	0.18	0.62	
360° Peak Turn Velocity (°/s) [Mean] **	361.32	63.78	249.12	77.21	<0.0001	0.88	
360° Peak Turn Velocity (°/s) [CoV]	0.05	0.02	0.06	0.03	0.09	0.61	

**Table 6:** Means, standard deviations, significance (*p*), independent AUC (I-AUC), and stepwise AUC (S-AUC) measures for 5×STS variables. Variable(s) with a single (*) were included in the stepwise regression.

	Young Adults		Middle-Aged Adults				
STS Variables	Mean	SD	Mean	SD	*p*	I-AUC	S-AUC
Total Duration (s) *	10.44	2.44	11.01	2.70	0.51	0.59	-
Sit to Stand-Duration (s) [mean] *	0.84	0.16	0.91	0.20	0.25	0.62	
Sit to Stand-Duration (s) [CoV]	0.13	0.06	0.13	0.06	0.95	0.49	
Sit to Stand-Lean Angle (°) [mean]	27.55	7.81	30.42	10.26	0.34	0.55	
Sit to Stand-Lean Angle (°) [CoV] *	0.14	0.08	0.11	0.06	0.22	0.61	
Stand to Sit-Duration (s) [mean] *	0.65	0.19	0.68	0.10	0.55	0.69	
Stand to Sit-Duration (s) [CoV]	0.20	0.17	0.18	0.14	0.64	0.54	
Stand to Sit-Lean Angle (°) [Mean]	24.52	8.99	25.68	9.61	0.71	0.52	
Stand to Sit-Lean Angle (°) [CoV] *	0.16	0.07	0.13	0.09	0.30	0.66	

**Table 7:** Means, standard deviations, significance (*p*), independent AUC (I-AUC), and stepwise AUC (S-AUC) measures for 5×STS variables. Variable(s) with a single (*) were included in the stepwise regression.

	Middle-Aged Adult		Older Adults				
STS Variables	Mean	SD	Mean	SD	*p*	I-AUC	S-AUC
Total Duration (s) *	11.01	2.70	12.77	3.00	0.07	0.68	0.86
Sit to Stand-Duration (s) [mean] **	0.91	0.20	1.03	0.21	0.07	0.69	
Sit to Stand-Duration (s) [CoV]	0.13	0.06	0.15	0.08	0.58	0.56	
Sit to Stand-Lean Angle (°) [mean] *	30.42	10.26	27.81	8.80	0.42	0.58	
Sit to Stand-Lean Angle (°) [CoV] **	0.11	0.06	0.17	0.09	0.03	0.69	
Stand to Sit-Duration (s) [mean]	0.68	0.10	0.71	0.13	0.44	0.55	
Stand to Sit-Duration (s) [CoV]	0.18	0.14	0.19	0.13	0.85	0.54	
Stand to Sit-Lean Angle (°) [mean]	25.68	9.61	23.12	8.73	0.41	0.58	
Stand to Sit-Lean Angle (°) [CoV] **	0.13	0.09	0.21	0.10	0.02	0.75	

**Table 8:** Top five AUC variables for each balance condition. The variable(s) with a solitary (*) denote a top five AUC variable for that condition, those with a double (**) were withheld in the stepwise regression model. Condition 1—eyes open, firm surface, Condition 2—eyes closed, firm surface, Condition 3—eyes open, compliant surface, Condition 4—eyes closed, compliant surface, I-AUC—independent AUC value, S-AUC—stepwise AUC value.

		Young Adults		Middle-Aged Adults				
Balance Variables	Condition	Mean	SD	Mean	SD	Interaction	I-AUC	S-AUC
Frequency Dispersion (Sagittal) (AD) *	1	0.69	0.04	0.70	0.04	0.16	0.64	-
Frequency Dispersion (Coronal) (AD) *		0.65	0.05	0.63	0.05	0.30	0.61	
Range (m/s^2^) *		0.46	0.11	0.48	0.13	0.82	0.57	
Mean Velocity (m/s) *		0.20	0.09	0.20	0.09	0.12	0.55	
Range (Sagittal) (m/s^2^) *		0.36	0.09	0.38	0.12	0.98	0.55	
Frequency Dispersion (Sagittal) (AD) **	2	0.68	0.04	0.71	0.03	0.16	0.71	0.74
95% Ellipse Radius (m/s^2^) *		1.60	0.66	1.79	0.49	0.81	0.65	
95% Ellipse Radius (°) *		1.60	0.66	1.79	0.49	0.81	0.65	
Centroidal Frequency (Coronal) (Hz) *		0.88	0.12	0.93	0.11	0.06	0.63	
Frequency Dispersion (Coronal) (AD) **		0.64	0.05	0.62	0.05	0.30	0.63	
Mean Velocity (Coronal) (m/s) **	3	0.19	0.09	0.13	0.09	0.09	0.74	0.74
Jerk (Sagittal) (m^2^/s^5^) *		5.80	4.09	7.15	3.56	0.16	0.65	
Path Length (Sagittal) (m/s^2^) *		9.64	3.06	10.98	2.85	0.15	0.65	
Centroidal Frequency (Hz) *		1.13	0.16	1.22	0.22	0.36	0.64	
Centroidal Frequency (Sagittal) (Hz) *		1.08	0.17	1.19	0.29	0.15	0.64	
Centroidal Frequency (Coronal) (Hz) **	4	0.89	0.16	0.98	0.13	0.06	0.67	0.67
Mean Velocity (Sagittal) (m/s) *		0.18	0.09	0.23	0.13	0.28	0.66	
Mean Velocity (m/s) *		0.28	0.17	0.31	0.11	0.12	0.66	
Frequency Dispersion (Sagittal) (AD) *		0.69	0.03	0.70	0.04	0.16	0.62	
Jerk (Sagittal) (m^2^/s^5^) *		16.54	12.04	18.36	10.49	0.16	0.62	

**Table 9:** Top five AUC variables for each balance condition. The variable(s) with a solitary (*) denote a top five AUC variable for that condition, those with a double (**) were withheld in the stepwise regression model. Condition 1—eyes open, firm surface, Condition 2—eyes closed, firm surface, Condition 3—eyes open, compliant surface, Condition 4—eyes closed, compliant surface, I-AUC—independent AUC value, S-AUC—stepwise AUC value.

		Middle-Aged Adults		Older Adults				
Balance Variables	Condition	Mean	SD	Mean	SD	Interaction	I-AUC	S-AUC
Frequency Dispersion (Sagittal) (AD) **	1	0.70	0.04	0.67	0.04	0.83	0.75	0.84
Mean Velocity (Coronal) (m/s) *		0.09	0.04	0.07	0.10	0.05	0.72	
Range (Coronal) (m/s^2^) **		0.28	0.10	0.23	0.15	0.18	0.65	
RMS Sway (Coronal) (m/s^2^) **		0.05	0.02	0.04	0.03	0.09	0.64	
Centroidal Frequency (Hz) *		1.01	0.14	0.94	0.21	0.09	0.63	
Frequency Dispersion (Sagittal) (AD) **	2	0.71	0.03	0.68	0.05	0.83	0.65	0.65
Range (Coronal) (m/s^2^) *		0.33	0.10	0.28	0.17	0.18	0.63	
Frequency Dispersion (Coronal) (AD) *		0.62	0.05	0.60	0.03	0.15	0.61	
RMS Sway (Sagittal) (°) *		0.50	0.24	0.54	0.17	0.01	0.61	
Range (Sagittal) (m/s^2^) *		0.08	0.04	0.09	0.03	<0.01	0.61	
95% Ellipse Axis 2 Radius (°) *	3	1.58	0.38	2.28	1.19	0.04	0.79	0.87
95% Ellipse Axis 2 Radius (m/s^2^) **		0.27	0.07	0.39	0.20	0.04	0.78	
RMS Sway (Sagittal) (m/s^2^) **		0.09	0.02	0.14	0.08	0.01	0.77	
RMS Sway (Sagittal) (°) **		0.52	0.13	0.80	0.50	0.01	0.77	
RMS Sway (°) *		0.77	0.15	1.10	0.59	0.03	0.77	
RMS Sway (Sagittal) (°) **	4	0.86	0.22	1.22	0.38	0.01	0.83	0.83
RMS Sway (Sagittal) (m/s^2^) *		0.15	0.04	0.21	0.07	0.01	0.82	
Range (Sagittal) (m/s^2^) *		0.83	0.22	1.33	0.69	<0.01	0.81	
RMS Sway (m/s^2^) *		0.21	0.04	0.29	0.11	0.03	0.78	
95% Ellipse Axis 2 Radius (°) *		2.47	0.58	3.48	1.27	0.04	0.78	

## Data Availability

The data presented in this study are available on request from the corresponding author. The data are not publicly available due to Institutional Review Board limitations.

## Appendix A

Appendix A provides definitions for the mobility variables listed throughout the text.

**Table A1:** Variable Definitions. Directional components for the measure in the sagittal and coronal (†), Mean and coefficient of variation measures (‡); Unilateral measures (i.e., separate left and right) (*); Multi-directional (¡): transverse, sagittal, and coronal; percent of the gait cycle time (%GCT); number (#).

APDM Gait Variable Definitions		
Variables	Units	Definition
Walking		
Cadence ‡	steps/min	The number of steps per minute, counting steps made by both feet
Stride Length ‡	m	The forward distance travelled by the foot during a gait cycle
Gait Cycle Duration ‡	s	The duration of a full gait cycle measured from the left foot’s initial contact to the next initial contact of the left foot
Step Duration ‡	s	The duration of a step measured as the period from initial contact of one foot to the next initial contact of the opposite foot. The handedness refers to the stepping foot.
Gait Speed ‡	m/s	The forward speed of the subject, measured as the forward distance traveled during the gait cycle divided by the gait cycle duration
Elevation at Mid-swing ‡ *	cm	The height of the foot sensor measured at mid-swing, relative to its start position while standing.
Circumduction ‡	cm	The maximum amount that the foot travels perpendicular to forward movement during an individual stride. Positive values indicate movement to the outside.
Lateral Step Variability *	cm	When considering three consecutive foot placements made by the same foot, this describes the variability of perpendicular deviations of the middle foot placement from the line connecting the first and the third. Positive values indicate movement to the outside.
Stance ‡	%GCT	The percentage of the gait cycle in which the foot is on the ground
Swing ‡	%GCT	The percentage of the gait cycle in which the foot is not on the ground
Single Limb Support ‡	%GCT	The percentage of the gait cycle in which one foot is on the ground
Double Limb Support ‡	%GCT	The percentage of the gait cycle in which both feet are on the ground
Terminal Double Support ‡	%GCT	The percentage of the gait cycle in which both feet are on the ground. The handedness refers to the foot that is in the forward position.
Toe Off Angle ‡	°	The angle of the foot as it leaves the floor at push off
Toe Out Angle ‡	°	The lateral angle of the foot during the stance phase, relative to the forward motion of the foot during the swing phase. Positive angles indicate an outward rotation of the toes.
Foot Strike Angle ‡	°	The angle of the foot at the point of initial contact
Lumbar		
Range of Motion ¡	°	The angular range of the lumbar spine in the designated plane
Trunk		
Range of Motion ¡	°	The angular range of the thoracic spine in the designated plane
APDM Turn Measures (180° and 360°)		
Turn Duration ‡	s	The duration of the turn
Turn Angle ‡	°	The rotational angle of the turn
Peak Turn Velocity ‡	°/s	The peak angular velocity of the turn
Turns Completed	#	Number of turns completed during the two-minute walk (180° turns only)
APDM Sit-to-Stand Variable Definitions		
Duration	s	The duration of the trial. Start and/or end timing delays are not included
Sit-to-Stand Variables		
Duration ‡	s	Duration of the sit-to-stand transition
Lean Angle ‡	°	The angular range of motion of the trunk during the sit-to-stand transition
Stand-to-Sit Variables		
Duration ‡	s	Duration of the stand-to-sit transition
Lean Angle ‡	°	The angular range of motion of the trunk during the stand-to-sit transition
APDM Postural Sway Variable Definitions		
Acceleration Variables		
Sway Area †	m^2^/s^4^	Sway area, computed as the area included in the sway trajectory per unit of time
Path Length †	m/s^2^	Total length of the sway path in the transverse plane
RMS Sway †	m/s^2^	The root mean square (RMS) of the sway angle in both the coronal and sagittal planes
Range †	m/s^2^	Total range of the sway path in the transverse plane
Mean Velocity †	m/s	Mean velocity of the sway path in the transverse plane
95% Ellipse Rotation †	radians	The area of an ellipse covering 95% of the sway angle in both the coronal and sagittal planes
Centroidal Frequency †	Hz	Frequency of sway from the centroid of the sway path’s power spectrum in the transverse plane
Frequency Dispersion †	AD	Frequency dispersion in the transverse plane
Jerk † ‡	m^2^/s^5^	Smoothness of sway from the time derivative of the sway path in the transverse plane (top view looking down)
Angle Variables		
Sway Area	°^2^	Sway area, computed as the area included in the sway trajectory per unit of time
95% Ellipse Rotation	radians	The area of an ellipse covering 95% of the sway angle in both the coronal and sagittal planes
RMS Sway †	°	The root mean square (RMS) of the sway angle in both the coronal and sagittal planes

## Appendix B

**Table A2:** Group by Condition post-hoc for gait variables demonstrating a significant interaction between Middle-aged Adults and Older Adults. ROM—range of motion, CoV—Coefficient of Variation, *p*—post-hoc significance.

		Middle-Aged Adult		Older Adult		
Variable	Condition	Mean	SD	Mean	SD	*p*
Trunk-Transverse ROM (°) [mean]	Normal	8.13	1.49	8.41	2.56	0.67
	Dual Task	8.99	1.73	8.34	2.72	0.38
	Fast	9.44	3.06	9.91	3.52	0.65
Lumbar-Sagittal ROM (°) [mean]	Normal	5.35	1.62	4.43	1.67	0.09
	Dual Task	4.85	1.39	4.30	1.55	0.24
	Fast	7.37	1.83	5.45	2.05	0.004
Lumbar-Coronal ROM (°) [CoV]	Normal	0.08	0.03	0.10	0.03	0.23
	Dual Task	0.08	0.02	0.11	0.04	0.008
	Fast	0.10	0.03	0.10	0.02	0.99
Toe Off Angle (°) [CoV]	Normal	0.04	0.01	0.05	0.02	0.01
	Dual Task	0.03	0.01	0.05	0.02	0.004
	Fast	0.03	0.01	0.03	0.01	0.09

**Table A3:** Condition by Group post-hoc for gait variables demonstrating a significant interaction between Middle-aged Adults and Older Adults. ROM—range of motion, CoV—Coefficient of Variation, *p*—post-hoc significance.

		Normal Walk		Dual Task Walk		Fast Walk		Normal vs. Dual Task	Normal vs. Fast	Dual Task vs. Fast
Variable	Group	Mean	SD	Mean	SD	Mean	SD	*p*	*p*	*p*
Trunk-Transverse ROM (°) [mean]	Middle-Age	8.13	1.49	8.99	1.73	9.44	3.06	0.44	0.15	0.79
	Older Adults	8.41	2.56	8.34	2.72	9.91	3.52	1.00	0.25	0.22
Lumbar-Sagittal ROM (°) [mean]	Middle-Age	5.35	1.62	4.85	1.39	7.37	1.83	0.60	0.001	0.0001
	Older Adults	4.43	1.67	4.30	1.55	5.45	2.05	0.97	0.17	0.11
Lumbar-Coronal ROM (°) [CoV]	Middle-Age	0.08	0.03	0.08	0.02	0.10	0.03	0.76	0.20	0.05
	Older Adults	0.10	0.03	0.11	0.04	0.10	0.02	0.66	0.97	0.80
Toe Off Angle (°) [CoV]	Middle-Age	0.04	0.01	0.03	0.01	0.03	0.01	0.93	0.21	0.38
	Older Adults	0.05	0.02	0.05	0.02	0.03	0.01	0.82	0.05	0.01

## Appendix C

**Table A4:** Group by Condition post-hoc for balance variables demonstrating a significant interaction between Middle-aged Adults and Older Adults. Means and SD are presented as well as post-hoc analysis *p*-values for each variable and condition. Condition 1: Eyes open, Firm Surface; Condition 2: Eyes Closed, Firm Surface; Condition 3: Eyes Open, Compliant Surface; Condition 4: Eyes Closed, Compliant Surface.

		Middle-Aged Adults		Older Adults		
Variable	Condition	Mean	SD	Mean	SD	*p*
95% Ellipse Axis 1 Radius (m/s^2^)	1	0.11	0.04	0.09	0.05	0.18
	2	0.13	0.04	0.11	0.07	0.56
	3	0.17	0.03	0.23	0.16	0.10
	4	0.29	0.06	0.39	0.18	0.02
95% Ellipse Axis 2 Radius (m/s^2^)	1	0.19	0.06	0.22	0.10	0.23
	2	0.22	0.10	0.23	0.07	0.64
	3	0.27	0.07	0.39	0.20	0.02
	4	0.42	0.10	0.59	0.21	0.004
Centroidal Frequency (Sagittal) (Hz)	1	0.88	0.18	0.82	0.23	0.37
	2	0.81	0.15	0.82	0.27	0.93
	3	1.19	0.29	1.00	0.22	0.03
	4	1.05	0.27	0.96	0.19	0.26
Mean Velocity (Coronal) (m/s)	1	0.09	0.04	0.07	0.10	0.54
	2	0.08	0.04	0.08	0.07	0.95
	3	0.13	0.09	0.16	0.11	0.37
	4	0.15	0.09	0.25	0.15	0.02
Range (m/s^2^)	1	0.48	0.13	0.48	0.17	0.91
	2	0.55	0.17	0.57	0.19	0.78
	3	0.76	0.12	1.07	0.79	0.09
	4	1.23	0.27	1.80	0.88	0.01
Range (Sagittal) (m/s^2^)	1	0.38	0.12	0.41	0.15	0.53
	2	0.43	0.17	0.48	0.15	0.40
	3	0.50	0.11	0.78	0.70	0.08
	4	0.83	0.22	1.33	0.69	0.004
RMS Sway (m/s^2^)	1	0.09	0.03	0.10	0.04	0.45
	2	0.10	0.04	0.11	0.03	0.77
	3	0.13	0.02	0.19	0.10	0.02
	4	0.21	0.04	0.29	0.11	0.01
RMS Sway (Sagittal) (m/s^2^)	1	0.07	0.02	0.08	0.04	0.32
	2	0.08	0.04	0.09	0.03	0.60
	3	0.09	0.02	0.14	0.08	0.02
	4	0.15	0.04	0.21	0.07	0.001
Sway Area (m^2^/s^4^)	1	0.07	0.04	0.07	0.06	0.98
	2	0.09	0.06	0.09	0.07	0.95
	3	0.15	0.05	0.36	0.59	0.11
	4	0.40	0.16	0.83	0.76	0.02
95% Ellipse Axis 1 Radius (°)	1	0.66	0.25	0.55	0.30	0.18
	2	0.74	0.26	0.68	0.41	0.57
	3	1.03	0.15	1.38	0.93	0.10
	4	1.72	0.34	2.33	1.03	0.02
95% Ellipse Axis 2 Radius (°)	1	1.12	0.34	1.30	0.57	0.22
	2	1.28	0.56	1.36	0.42	0.63
	3	1.58	0.38	2.28	1.19	0.02
	4	2.47	0.58	3.48	1.27	0.004
RMS Sway (°)	1	0.54	0.16	0.59	0.24	0.43
	2	0.61	0.23	0.63	0.20	0.75
	3	0.77	0.15	1.10	0.59	0.02
	4	1.24	0.26	1.72	0.65	0.005
RMS Sway (Sagittal) (°)	1	0.44	0.14	0.50	0.22	0.31
	2	0.50	0.24	0.54	0.17	0.58
	3	0.52	0.13	0.80	0.50	0.02
	4	0.86	0.22	1.22	0.38	0.001
Sway Area (°^2^)	1	2.49	1.53	2.49	2.01	1.00
	2	3.13	1.96	3.09	2.50	0.96
	3	5.13	1.56	12.72	20.99	0.12
	4	13.83	5.65	28.91	26.23	0.02

**Table A5:** Condition by Group post-hoc for balance variables demonstrating a significant interaction between Middle-aged Adults and Older Adults. Means and SD are presented as well as post-hoc analysis *p*-values for each variable and condition comparison. Condition 1: Eyes open, Firm Surface; Condition 2: Eyes Closed, Firm Surface; Condition 3: Eyes Open, Compliant Surface; Condition 4: Eyes Closed, Compliant Surface.

Condition by Group		Condition 1		Condition 2		Condition 3		Condition 4		Post-hoc Comparisons					
Variables	Group	Mean	SD	Mean	SD	Mean	SD	Mean	SD	1-2	1-3	1-4	2-3	2-4	3-4
95% Ellipse Axis 1 Radius (m/s^2^)	Middle-Aged	0.11	0.04	0.13	0.04	0.17	0.03	0.29	0.06	0.79	<0.002	<0.001	0.004	<0.001	<0.001
	Older Adults	0.09	0.05	0.11	0.07	0.23	0.16	0.39	0.18	0.94	0.003	<0.001	0.014	<0.001	0.0012
95% Ellipse Axis 2 Radius (m/s^2^)	Middle-Aged	0.19	0.06	0.22	0.10	0.27	0.07	0.42	0.10	0.71	0.015	<0.001	0.194	<0.001	<0.001
	Older Adults	0.22	0.10	0.23	0.07	0.39	0.20	0.59	0.21	1.00	0.006	<0.0001	0.01	<0.001	0.001
Centroidal Frequency (Sagittal) (Hz)	Middle-Aged	0.88	0.18	0.81	0.15	1.19	0.29	1.05	0.27	0.79	<0.001	0.104	<0.001	0.01	0.25
	Older Adults	0.82	0.23	0.82	0.27	1.00	0.22	0.96	0.19	1.00	0.084	0.337	0.08	0.328	0.95
Mean Velocity (Coronal) (m/s)	Middle-Aged	0.09	0.04	0.08	0.04	0.13	0.09	0.15	0.09	0.96	0.246	0.026	0.09	0.007	0.75
	Older Adults	0.07	0.10	0.08	0.07	0.16	0.11	0.25	0.15	1.00	0.061	<0.001	0.09	<0.001	0.08
Range (m/s^2^)	Middle-Aged	0.48	0.13	0.55	0.17	0.76	0.12	1.23	0.27	0.59	<0.001	<0.001	0.004	<0.001	<0.001
	Older Adults	0.48	0.17	0.57	0.19	1.07	0.79	1.8	0.88	0.97	0.011	<0.001	0.037	<0.001	0.003
Range (Sagittal) (m/s^2^)	Middle-Aged	0.38	0.12	0.43	0.17	0.50	0.11	0.83	0.22	0.70	0.078	<0.001	0.53	<0.001	<0.001
	Older Adults	0.41	0.15	0.48	0.15	0.78	0.70	1.33	0.69	0.97	0.076	<0.001	0.20	<0.001	0.009
RMS Sway (m/s^2^)	Middle-Aged	0.09	0.03	0.10	0.04	0.13	0.02	0.21	0.04	0.65	0.002	<0.001	0.06	<0.001	<0.001
	Older Adults	0.10	0.04	0.11	0.03	0.19	0.10	0.29	0.11	0.99	0.003	<0.001	0.009	<0.001	<0.001
RMS Sway (Sagittal) (m/s^2^)	Middle-Aged	0.07	0.02	0.08	0.04	0.09	0.02	0.15	0.04	0.74	0.494	<0.001	0.98	<0.001	<0.001
	Older Adults	0.08	0.04	0.09	0.03	0.14	0.08	0.21	0.07	0.99	0.03	<0.001	0.07	<0.001	0.002
Sway Area (m^2^/s^4^)	Middle-Aged	0.07	0.04	0.09	0.06	0.15	0.05	0.40	0.16	0.92	0.048	<0.001	0.19	<0.001	<0.001
	Older Adults	0.07	0.06	0.09	0.07	0.36	0.59	0.83	0.76	1.00	0.194	<0.001	0.24	<0.001	0.019
95% Ellipse Axis 1 Radius (°)	Middle-Aged	0.66	0.25	0.74	0.26	1.03	0.15	1.72	0.34	0.79	<0.002	<0.001	0.0043	<0.001	<0.001
	Older Adults	0.55	0.30	0.68	0.41	1.38	0.93	2.33	1.03	0.94	0.002	<0.001	0.014	<0.001	0.001
95% Ellipse Axis 2 Radius (°)	Middle-Aged	1.12	0.34	1.28	0.56	1.58	0.38	2.47	0.58	0.71	0.016	<0.001	0.198	<0.001	<0.001
	Older Adults	1.30	0.57	1.36	0.42	2.28	1.19	3.48	1.27	1.00	0.006	<0.001	0.011	<0.001	0.001
RMS Sway (°)	Middle-Aged	0.54	0.16	0.61	0.23	0.77	0.15	1.24	0.26	0.65	0.002	<0.001	0.065	<0.001	<0.001
	Older Adults	0.59	0.24	0.63	0.20	1.10	0.59	1.72	0.65	0.99	0.003	<0.001	0.009	<0.001	<0.009
RMS Sway (Sagittal) (°)	Middle-Aged	0.44	0.14	0.50	0.24	0.52	0.13	0.86	0.22	0.74	0.497	<0.001	0.98	<0.001	<0.001
	Older Adults	0.50	0.22	0.54	0.17	0.80	0.50	1.22	0.38	0.99	0.03	<0.001	0.07	<0.001	0.003
Sway Area (°^2^)	Middle-Aged	2.49	1.53	3.13	1.96	5.13	1.56	13.83	5.65	0.92	0.051	<0.001	0.202	<0.001	<0.001
	Older Adults	2.49	2.01	3.09	2.50	12.72	20.99	28.91	26.23	1.00	0.19	<0.001	0.237	<0.001	0.021
